# Predicting the risk for lymphoma development in Sjogren syndrome: An easy tool for clinical use

**DOI:** 10.1097/MD.0000000000003766

**Published:** 2016-06-24

**Authors:** Sofia Fragkioudaki, Clio P. Mavragani, Haralampos M. Moutsopoulos

**Affiliations:** a Department of Physiology; b Department of Pathophysiology; c Joint Academic Rheumatology Program, School of Medicine, National and Kapodistrian University of Athens, Athens, Greece.

**Keywords:** adverse predictors, non-Hodgkin lymphoma, Sjogren syndrome

## Abstract

The heightened risk of non-Hodgkin lymphoma (NHL) development in primary Sjogren syndrome (SS) is well established. Several adverse clinical and laboratory predictors have been described. In the current work, we aimed to formulate a predictive score for NHL development, based on clinical, serological, and histopathological findings at the time of SS diagnosis. In the present case–control study of 381 primary SS patients and 92 primary SS patients with concomitant NHL, clinical, serological, and histopathological variables at the time of SS diagnosis were retrospectively recorded. For the identification of predictors for NHL development univariate and multivariate models were constructed. Salivary gland enlargement (SGE), lymphadenopathy, Raynaud phenomenon, anti-Ro/SSA or/and anti-La/SSB autoantibodies, rheumatoid factor (RF) positivity, monoclonal gammopathy, and C4 hypocomplementemia were shown to be independent predictors for NHL development. On the basis of the number of independent risk factors identified, a predictive risk score for NHL development was formulated. Thus, patients presenting with ≤2 risk factors had a 3.8% probability of NHL development, those with 3 to 6 risk factors 39.9% (OR (95%CI): 16.6 [6.5–42.5], *P* < 0.05), while in the presence of all 7 risk factors the corresponding probability reached 100% (OR [95%CI]: 210.0 [10.0–4412.9], *P* < 0.0001). In conclusion, an easy to use diagnostic scoring tool for NHL development in the context of SS is presented. This model is highly significant for the design of early therapeutic interventions in high risk SS patients for NHL development.

## Abstract 2

Supplemental Digital Content is available in the text

## 1 Introduction

Sjogren syndrome (SS) is a common systemic autoimmune disease usually confined in the exocrine glands (mainly salivary and lachrymal), leading to desiccation of oral and ocular mucosal tissues. Nevertheless, systemic manifestations can arise in a proportion of SS individuals^[1]^ and B-cell non-Hodgkin lymphoma (NHL) development represents a severe complication, afflicting approximately 5% of patients.^[2]^ The risk of NHL occurrence in the setting of SS, the highest among systemic autoimmune diseases,^[3,4]^ has been previously estimated to be 7- to 19-fold higher compared to the general population.^[3–9]^ Although mucosa associated lymphoid tissue (MALT) mainly in the salivary glands is the prominent histological lymphoma type with a 1000-fold increased risk^[4]^ among primary SS patients,^[2,10]^ more aggressive subtypes including diffuse large B-cell lymphomas can also occur.^[2,11]^

Lymphomagenesis in the setting of autoimmunity and particularly of SS is considered a multifactorial process, not entirely elucidated yet. Genetic aberrations, including chromosomal translocations,^[12]^ mutations of the tumor suppressor gene p53,^[13]^ and polymorphisms of molecules with regulatory role in both innate and adaptive immune activation pathways^[14,15]^ have been so far implicated. Moreover, according to previous studies, clinical features at disease presentation, such as persistent salivary gland enlargement (SGE)^[16]^ and palpable purpura,^[16,17]^ laboratory abnormalities, like lymphopenia, monoclonal type II cryoglobulinemia, and hypocomplementemia^[16–18]^ as well as intense lymphocytic infiltrations^[19]^ and germinal center formation^[20]^ in minor salivary gland (MSG) biopsies, have been identified as adverse predictors for SS-related NHL development. As a result, at their first evaluation, SS patients can be classified into separate subsets with distinct likelihood for lymphoma development.

The current study aimed to create a predictive tool in clinical practice for SS-related NHL development, based on clinical, hematological, serological, and histopathological features, observed early at disease diagnosis. Prompt diagnosis would allow early therapeutic intervention with the ultimate goal to decelerate the progression of benign to malignant lymphoproliferation.

## 2 Methods

### 2.1 Study cohort

Medical records of 381 primary SS patients (SS) without and 92 SS patients with concomitant NHL (SS NHL), fulfilling the revised European/American International classification criteria for SS^[21]^ and derived from the Department of Pathophysiology, University of Athens, a personal patient collection of Prof. HMM, and the Department of Rheumatology in “G Gennimatas” General Hospital, were retrospectively evaluated. Patients with SS secondary to other systemic autoimmune diseases were excluded. A total of 83.7% of the entire patient group (both SS and SS NHL) had undergone MSG biopsy (63.9% had positive MSG, defined as focus score ≥1) and 92.6% were evaluated for anti-Ro/SSA or/and anti-La/SSB status (74.4% were anti-Ro/SSA or/and anti-La/SSB positive). Among 92 SS NHL patients, 73 had MALT and 19 non-MALT lymphoma. The latter included 12 diffuse large B-cell lymphoma (2 of which derived from MALT lymphoma transformation), 4 nodal marginal zone lymphoma, 2 small lymphocytic lymphoma, and 1 T-cell lymphoma. Informed consent was waived due to retrospective nature of the study.

### 2.2 Demographic, clinical, and laboratory evaluation

Demographic, clinical, and laboratory data, at the time of SS diagnosis, were collected through an extensive clinical chart review. Information regarding the presence of glandular manifestations such as oral, ocular, skin and upper respiratory tract dryness, SGE, as well as ocular (abnormal Schirmer test ≤5 mm/5 minutes and ocular dye score ≥4) and oral (unstimulated salivary flow ≤1.5 mL/15 minutes) signs was obtained. Systemic features such as musculoskeletal discomfort, including myalgias, arthralgias and arthritis, Raynaud phenomenon, palpable purpura, peripheral nervous system (PNS) involvement based on electrophysiological studies, lymphadenopathy, splenomegaly and histologically proven interstitial renal disease, glomerulonephritis, autoimmune hepatitis, or primary biliary cirrhosis were recorded. In the SS NHL group, the histological subtype of lymphoma was also documented.

Laboratory data included hematological features, such as leukocyte and platelet number and hemoglobulin levels, as well as serological characteristics such as hypergammaglobulinemia and monoclonal gammopathy, autoantibodies (antinuclear antibodies, anti-Ro/SSA, anti-La/SSB antibodies, rheumatoid factor [RF], antimitochondrial, and anti-thyroid), cryoglobulins, and C3 and C4 complement protein levels. Leukopenia was defined as white blood cells number <4000/μL, lymphocytopenia as lymphocytes number <1000/μL, thrombocytopenia as platelets number <250,000/μL, anemia as hemoglobulin levels <12 g/dL, C3 and C4 hypocomplementemia as levels <90 and 20 mg/dL, respectively, and RF positivity as levels >20 IU/mL.

At the level of MSG tissue, the extent of lymphocytic infiltration, evaluated using Tarpley and focus scores,^[21]^ germinal center formation, and the presence of monoclonality (as previously described^[22]^) was also recorded. For continuous variables such as Tarpley and focus scores, their median values were chosen as the cut-off level.

### 2.3 Statistical analysis

**Figure 1:**
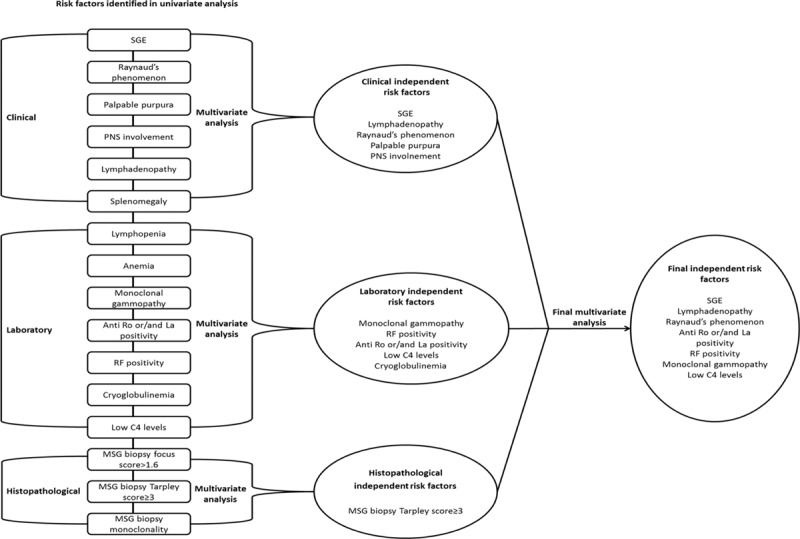
Identification of independent risk factors for NHL development in 2 steps. First, risk factors identified to be statistically significant in the univariate analysis were analyzed in 3 separate multivariate models and independent clinical, laboratory, and histopathological risk factors for NHL development were determined. Second, a final multivariate logistic regression analysis including all independent risk factors, revealed in the 3 separate multivariate models, was performed. MSG = minor salivary gland, NHL = non-Hodgkin lymphoma, PNS = peripheral nervous system, RF = rheumatoid factor, SGE = salivary gland enlargement.

Comparison of qualitative and quantitative features between SS patients with and without NHL was performed with Fisher exact/Chi-square test and Mann–Whitney *U* tests respectively using SPSS software 21.0. For data analysis, univariate and multivariate logistic regression models were implemented. We first classified predictors for lymphoma development into 3 major groups including clinical, laboratory, and histopathological features, respectively. Next, 3 separate multivariate models were constructed for each group, each of which included only those parameters found to be significant in univariate analysis. In order to explore whether the identified variables are highly correlated each other, a principal component analysis was performed as previously described.^[23]^ Last, a final multivariate model, including the independent predictors found to be significant in the 3 separate models was built (Fig. 1). A *P*-value <0.05 and 0.1 for univariate and multivariate analysis was considered statistically significant, respectively. The final list of independent predictors—identified in the last step—was used to calculate the relative risk for NHL according to the equation:

Risk = [exp(βl × xli + … + βp × xpi)]/{1 + [exp(βl × xli + … + βp × xpi)]}

In this equation, β1 to βp are the regression coefficients of the independent features, while xli to xpi are the values corresponding to the independent risk factors for a particular patient.

Measures of calibration (Hosmer–Lemeshow statistics) and discrimination (receiver operating characteristic statistic) were calculated to evaluate the overall performance of the predictive model. Binary logistic regression was used to calculate the prognostic probability of developing SS related NHL based on the number of risk factors (identified in the final step of multivariate analysis) presenting at the time of SS diagnosis and odds ratios (ORs) with 95% confidence intervals (CIs) were calculated. Analyses were performed by Graph Pad Prism 5.00 and SPSS software 21.0.

## 3 Results

### 3.1 Demographic data

Demographic data for the SS and SS NHL groups are shown in Table 1. The mean age at disease diagnosis of the SS and SS NHL cohort was 51.6 ± 13.2 and 50.3 ± 13.4, respectively, while the female-to-male ratio was 17:1 and 14:1, respectively. The corresponding ages for the SS MALT and non-MALT groups were 49.9 ± 12.7 and 52.1 ± 16.2, respectively. No significant differences between groups were detected.

**Table 1:**
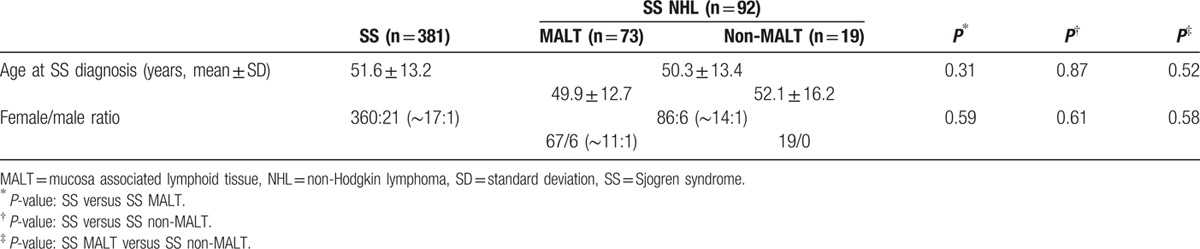
Demographic data of the study cohort.

### 3.2 Clinical, hematological, serological, and histopathological features in SS and SS NHL groups

In Tables 2 and 3, the prevalence of clinical and laboratory manifestations at disease onset in SS patients with and without NHL is presented (univariate analysis). The 2 groups had similar rates of symptoms related to exocrine dysfunction (oral, ocular, skin, and upper respiratory system dryness), of musculoskeletal discomfort, including arthritis, as well as renal and liver involvement. In contrary, compared to the SS group, SS NHL patients exhibited increased frequency of Raynaud phenomenon (37.0% vs 23.9%, *P* = 0.01), SGE (64.1% vs 21.5%, *P* < 0.001), palpable purpura (42.4% vs 12.1%, *P* < 0.001), lymphadenopathy (44.6% vs 10.2%, *P* < 0.001), splenomegaly (8.7% vs 1.1%, *P* < 0.001), and PNS involvement (8.7% vs 2.4%, *P* = 0.01). Additionally, SS NHL occurrence was associated with lymphopenia (28.3% vs 11.6%, *P* < 0.001), anemia (46.7% vs 23.9%, *P* < 0.001), RF positivity (85.4% vs 52.4%, *P* < 0.001), anti-Ro/SSA or/and anti-La/SSB positivity (91.2% vs 70.0%, *P* < 0.001), monoclonal gammopathy (23.3% vs 5.0%, *P* < 0.001), as well as cryoglobulinemia (32.1% vs 6.5%, *P* < 0.001) and low C4 complement levels (80.9% vs 48.1%, *P* < 0.001). In regard to the histopathological features on the initial diagnostic salivary gland biopsy, an MSG focus score more than 1.6 (71.4% vs 42.0%, *P* < 0.001), a Tarpley score ≥3 (68.5% vs 38.5%, *P* < 0.001), as well as the presence of monoclonality in MSG tissues (50.0% vs 10.7%, *P* = 0.003) have been all found to occur more frequently in the SS NHL compared to the SS group.

**Table 2:**
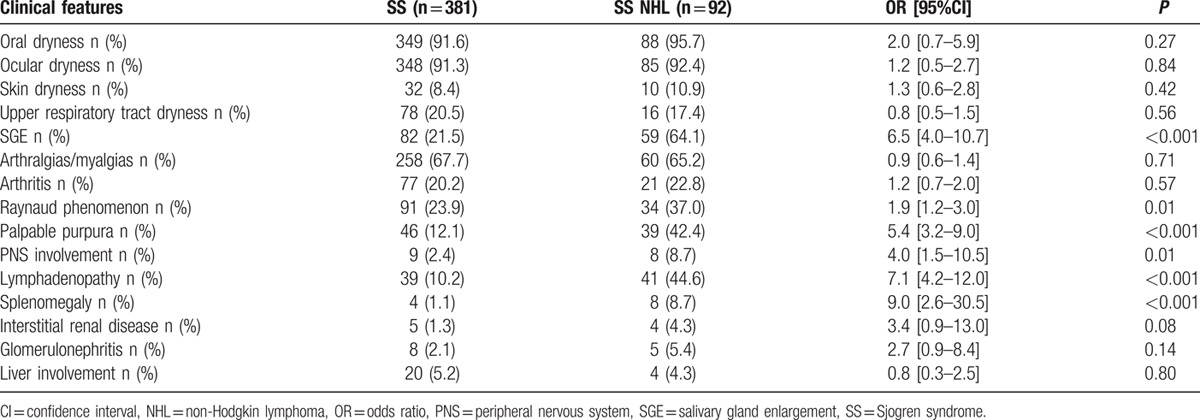
Prevalence of clinical features, at time of diagnosis, in SS patients with and without non-Hodgkin lymphoma (univariate analysis).

**Table 3:**
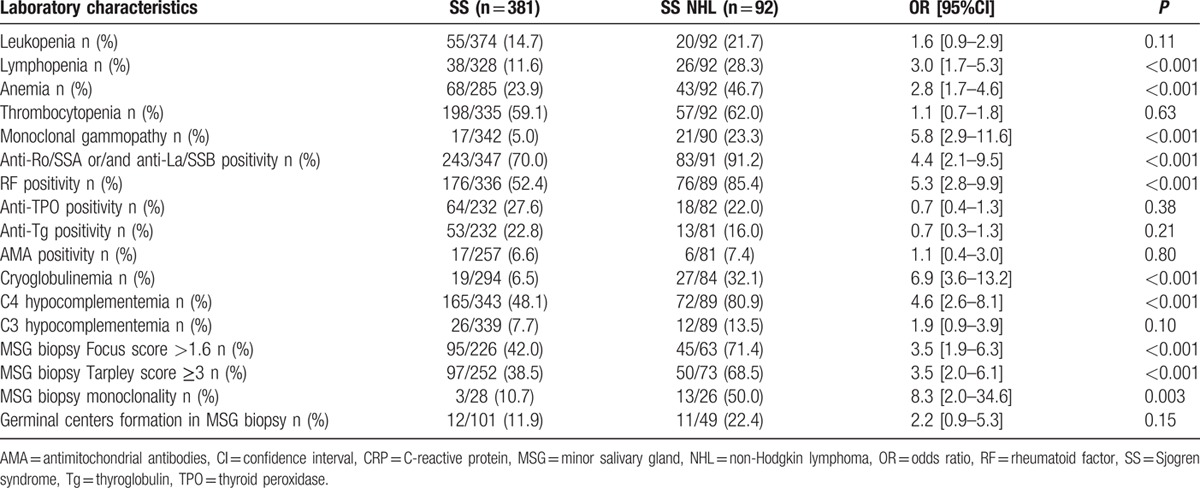
Comparison of hematological, serological, and histopathological characteristics between SS patient groups with and without non-Hodgkin lymphoma, at time of diagnosis (univariate analysis).

### 3.3 Independent risk factors for NHL development

Table 4 displays the results of the 3 separate multivariate models on clinical, serological, and histopathological parameters. Clinical variables found to be independently associated with NHL included SGE, lymphadenopathy, palpable purpura, PNS involvement, and Raynaud phenomenon (OR [95%CI]: 5.3 [3.1–9.0], 4.5 [2.5–8.1], 3.3 [1.8–6.1], 3.0 [0.9–10.5], and 1.6 [0.9–2.9], respectively). Serological and histopathological features independently predicting NHL development were RF, anti-Ro/SSA or/and anti-La/SSB positivity, monoclonal gammopathy, C4 hypocomplementemia, cryoglobulinemia, and Tarpley score in the MSG biopsy ≥3. (OR [95%CI]: 3.4 [1.5–7.3], 7.5 [2.2–25.5], 4.76 [1.6–13.9], 2.9 [1.5–5.9], 2.7 [1.2–6.3], and 5.8 [2.7–12.5], respectively).

**Table 4:**
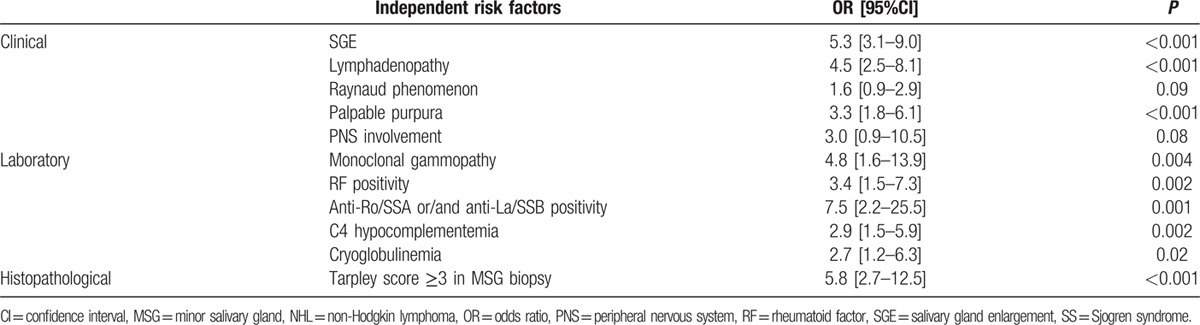
Independent clinical, laboratory, and histopathological risk factors for SS-related non-Hodgkin lymphoma development, identified by 3 distinct multivariate analysis.

To test whether the identified variables are highly associated each other inflating the results of the multivariate model, a principal component analysis was performed. Supplementary Table 1, displays the variance of each of the principal component scores amongst the 473 patients studied showing the proportion of variance in the dataset explained by each of the principal components. Since the first 3 principal components do not contribute to the overall variability significantly (PC1:23.8%, PC2: 14.9%, and PC3:12.8%), all individual variables were included in the final prediction model.

Thus, all the independent predictors resulting from the three separate multivariate models (clinical, laboratory, and histopathological) were subsequently included in a final multivariate model with SGE, lymphadenopathy, Raynaud phenomenon, anti-Ro/SSA or/and anti-La/SSB positivity, RF positivity, monoclonal gammopathy, and C4 hypocomplementemia being identified as independent predictors for NHL development: (OR [95%]: 4.3 [2.0–9.1], 4.2 [1.8–9.9], 2.3 [1.0–5.2], 3.8 [1.1–13.4], 3.7 [1.4–10.0], 3.2 [1.0–9.8], 3.0 [1.3–6.8]) (Table 5).

**Table 5:**
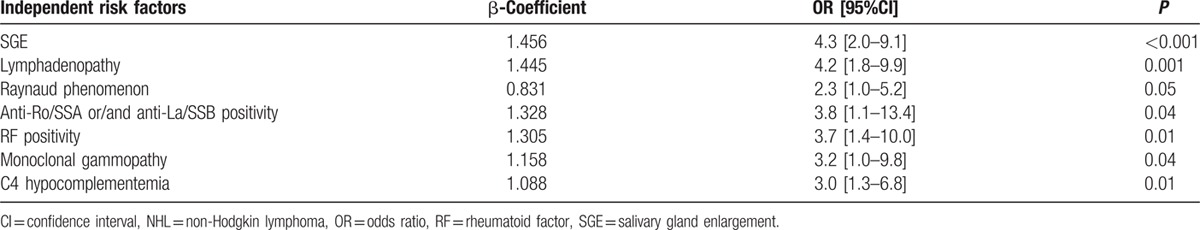
Final independent risk factors for non-Hodgkin lymphoma development, after multivariate analysis of all variables found to be significant in separate multivariate models.

### 3.4 Prediction score for SS NHL development

Based on the results of the logistic regression analysis a predictive model was formulated. In this model, the relative risk for NHL development was calculated for each patient according to the following equation, as previously described^[24–26]^:

Risk = EXP[SGE^∗^(1.456) + Raynaud phenomenon^∗^(0.831) + lymphadenopathy^∗^(1.445) + monoclonal gammopathy^∗^(1.158) + RF positivity^∗^(1.305) + C4 hypocomplementemia^∗^(1.088) + anti-Ro/SSA or/and La/SSB positivity^∗^(1.328)]/{1 + EXP [SGE^∗^(1.456) + Raynaud phenomenon^∗^(0.831) + lymphadenopathy^∗^(1.445) + monoclonal gammopathy^∗^(1.158) + RF positivity^∗^(1.305) + C4 hypocomplementemia^∗^(1.088) + anti-Ro/SSA or/and La/SSB positivity^∗^(1.328)]}

**Figure 2:**
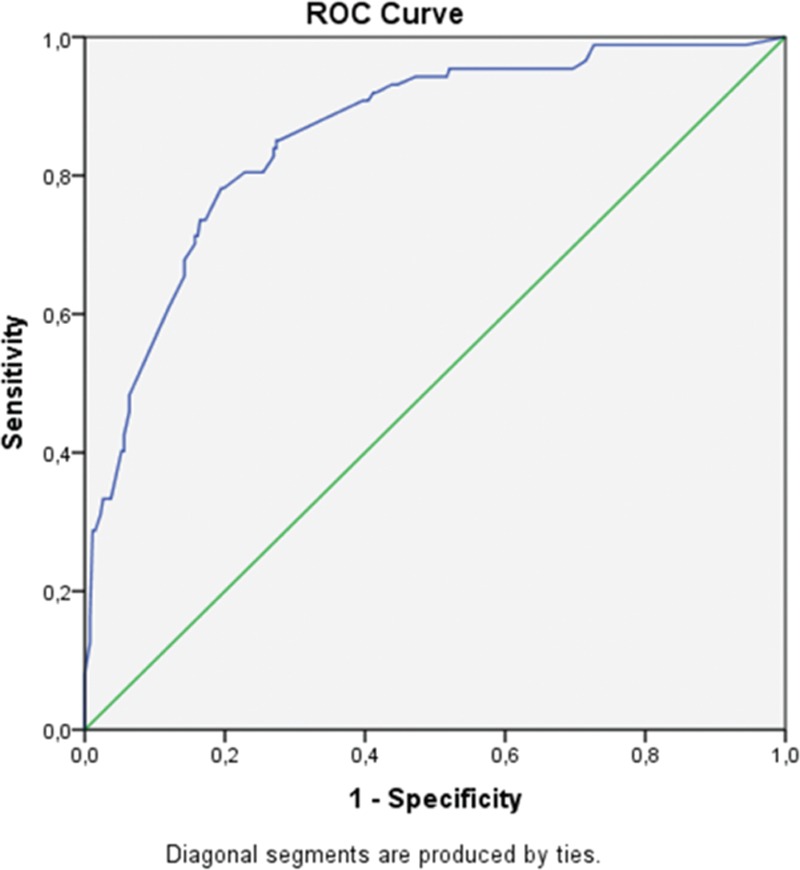
The performance evaluation of the predictive model for NHL development with the formation of ROC curves. The AUC was 0.9 (95%CI: 0.8–0.9, *P* < 0.001). AUC = area under the curve, CI = confidence interval, NHL = non-Hodgkin lymphoma, ROC = receiver operating characteristic.

In these formulas, binary variables were coded as follows—SGE: presence = 1, absence = 0; Raynaud phenomenon: presence = 1, absence = 0; lymphadenopathy: presence = 1, absence = 0; monoclonal gammopathy: presence = 1, absence = 0; RF positivity: presence = 1, absence = 0; C4 hypocomplementemia: presence = 1, absence = 0; and anti-Ro/SSA and/or La/SSB positivity: presence = 1, absence = 0. When receiver operating characteristic curves for the predictive model were fitted, the area under the curve was 0.9, 95%CI: 0.8 to 0.9, *P* < 0.001 (Fig. 2). Hosmer–Lemeshow goodness-of-fit statistics were 4.8, *P* = 0.78.

**Figure 3:**
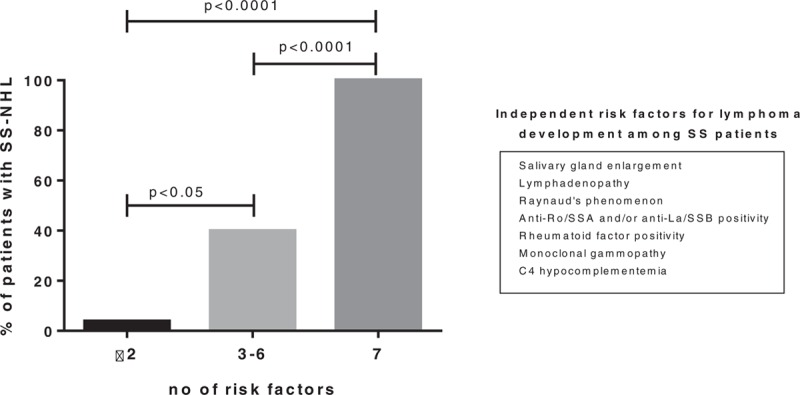
The probability of NHL development among SS patients was estimated on the basis of the number of independent risk factors. The probability of NHL development was 3.8% for patients presenting with ≤2 risk factors, 39.9% for those displaying 3 to 6 risk factors, and 100% in the presence of all 7 risk factors. The OR along with the corresponding CI and *P*-values for NHL development in the presence of all 7 risk factors were 210.0 (10.0–4412.9), *P* < 0.0001 compared to those with 2 or less risk factors. The corresponding values in the presence of 3 to 6 risk factors were 16.6 (6.5–42.5), *P* < 0.05 in comparison with those with 2 or less risk factors. CI = confidence interval, NHL = non-Hodgkin lymphoma, OR = odds ratio, SS = Sjogren syndrome.

Binary logistic regression was used to calculate the predicted probability of NHL development. Only patients with full data available (325 patients out of 373, 87% of the initial cohort) were analyzed. In the absence of those 7 risk factors, none of the SS patients in the cohort had lymphoma. Patients presenting with ≤2 had a 3.8% probability of NHL development. The probability of NHL development in the presence of 3 to 6 risk factors was 39.9%, while in the presence of all 7 risk factors was 100%. The ORs along with the corresponding CIs and *P*-values for NHL development in the presence of all 7 risk factors were 210.0 (10.0–4412.9), *P* < 0.0001 compared to those with 2 or less risk factors. The corresponding values in the presence of 3 to 6 risk factors were 16.6 (6.5–42.5), *P* < 0.05 in comparison with patients presenting with 2 or less risk factors (Fig. 3).

## 4 Discussion

Lymphoid malignancy is an undesired complication, encountered in a considerable proportion of SS patients, who have the highest risk compared to patients with other systemic autoimmune disorders.^[3,4,8]^ In the current study, we identified a predictive model for NHL development, based on the initial clinical, laboratory, and histopathological evaluation of SS patients. Clinical manifestations such as SGE, lymphadenopathy, palpable purpura, peripheral neuropathy and Raynaud phenomenon, serological features including RF and anti-Ro/SSA or/and anti-La/SSB autoantibodies positivity, monoclonal gammopathy, C4 hypocomplementemia, and cryoglobulinemia, as well as extensive lymphocytic infiltration in MSG biopsy (Tarpley score ≥3) were found to be associated with NHL development. In a last step multivariate model, taken into consideration all the previously identified predictors, only SGE, lymphadenopathy, Raynaud phenomenon, anti-Ro/SSA or/and anti-La/SSB as well as RF positivity, monoclonal gammopathy, and C4 hypocomplementemia were determined as independent adverse predictors for NHL development. A predictive score for NHL development was formulated based on the number of independent risk factors. The probability of NHL development was 3.8% for patients presenting with ≤2 risk factors, 39.9% for those having 3 to 6 risk factors and reached 100% in the presence of all 7 risk factors.

Our current findings are in accord with previously published data supporting several clinical and laboratory variables as predictors of NHL development. Clinical features such as SGE, lymphadenopathy,^[2,16,18,27–30]^ as well as manifestations related to immunocomplexes deposition, including palpable purpura^[5,16,17]^ and peripheral neuropathy^[2,31]^ have been consistently identified as determinants of severe SS phenotypic variants. The emergence of Raynaud phenomenon as an independent adverse predictor for NHL development is in accord with previous observations in a US nationwide study.^[32]^ Of interest, the presence of anticentromere antibodies in a subset of SS individuals has been previously associated with both Raynaud phenomenon and heightened NHL risk.^[33]^ Unfortunately, this association was not explored in this study, due to the limited availability of anticentromere antibodies autoantibody data.

In line with previous findings revealing associations between anti-Ro/SSA and/or anti-La/SSB autoantibodies either with systemic manifestations associated with NHL development^[34–36]^ or with NHL development itself,^[37]^ we also found that antibodies against these ribonucleoproteinic complexes are an independent predictor for NHL development. In the same context, monoclonal gammopathy,^[29,38,39]^ hypocomplementemia, and cryoglobulinemia^[5,9,16,18,28,29,34,37,40]^ previously associated with malignant transformation, possibly as a result of excessive B-cell activation, have also been shown to be independently related to NHL occurrence and increased mortality.^[5,40,41]^ Monoclonal mixed cryoprecipitates, reported as a detrimental prognostic factor for SS-related lymphomagenesis,^[17]^ contain monoclonal RF, secreted by a subset of malignant B-cells derived by clonally expanded B cells exhibiting RF activity,^[42]^ which has been emerged as an independent predictor for NHL in both Greek and French cohorts.^[43]^

In relation to histopathological variables, we have also observed an association between NHL development with the density and monoclonality of lymphocytic infiltrations as well as a positive trend towards germinal center formation. Multivariate analysis revealed Tarpley score ≥3 as an independent risk factor for lymphoma development, in accord with previous observations.^[19,44]^ The presence of monoclonality^[22,45]^ as well as the formation of germinal centers^[20]^ may also alert for future lymphoma development, as previously proposed, though they were not identified as independent predictors in the current work, possibly due to the limited number of patients.

The identified independent predictors for NHL development in the setting of SS, from our group and others, including autoantibody production and manifestations attributed to immunocomplexes formation and activation of the classical component pathway leading to hypocomplementemia, point B-cell activation as a central pathogenetic mechanism of SS-related lymphomagenesis. It is of interest that these adverse predictors are present early, as soon as the diagnosis of SS is made, implying that a distinct genetic background might determine low and high risk SS subtypes. In support of this hypothesis, genetic alterations related to B cell activation, such as variants of B-cell activating factor, a survival factor for B lymphocytes,^[14]^ tumor necrosis factor alpha-induced protein 3, a gatekeeper of NFKB activation,^[15]^ and the His159Tyr of the B-cell activating factor receptor previously shown to enhance alternate NFKB signaling^[46,47]^ and immunoglobulin production,^[46]^ are implicated in the pathogenesis of SS MALT lymphoma.^[47]^ Other molecules associated with B lymphocytes proliferation and organization in lymphoid tissues, such as Fms-like tyrosine kinase 3 ligand^[48]^ and chemokine C-X-C motif ligand 13,^[49]^ have also been proposed as serum biomarkers of lymphoma in the setting of SS. However, the entire mechanisms leading from benign proliferation to malignant transformation remain to be elucidated.

One of the major limitations of the current study could be considered the relatively small number of SS-NHL cases, though they consist one of the largest currently available SS-lymphoma databases, given their rarity and the unrecognized diagnosis in the general population. The relatively low number of patients could also account for the lack of retention of monoclonality at the level of salivary gland tissue as independent predictor of lymphoma development in the multivariate model. On the other hand, the clustering of both MALT and non-MALT NHL cases in a whole group did not allow the identification of distinct predictors between the 2 lymphoma subtypes which are characterized by separate pathogenetic events. Further multicenter efforts including larger number of patients could both clarify this issue and validate the currently proposed prediction algorithm.

Identification of a high risk phenotype for lymphoma development at the time of SS diagnosis has been long appreciated as a major diagnostic challenge. Although individual clinical and laboratory parameters have been identified in the past as predictors of NHL in the context of SS, for the first time, we developed an easy to use risk assessment tool in everyday clinical practice, based on combinations of independent adverse predictors, allowing at the same time the design of early preventative therapeutic strategies in high risk SS patients for NHL development.

## Supplementary Material

Supplemental Digital Content
