# Supplementary material for: Predicting the risk for lymphoma development in Sjogren syndrome: An easy tool for clinical use
Source: Medicine (Baltimore). 2016 Jun 24;95(25):e3766. doi: 10.1097/MD.0000000000003766 (PMC4998301; doi:10.1097/MD.0000000000003766)
Supplement: Supplemental Digital Content [file medi-95-e3766-s001.doc]

**Supplementary table 1**

| **Total Variance Explained^a^** | | | | | | |
| --- | --- | --- | --- | --- | --- | --- |
| Component | Initial Eigenvalues | | | Extraction Sums of Squared Loadings | | |
|  | Total | % of Variance | Cumulative % | Total | % of Variance | Cumulative % |
| 1 | 2,620 | 23,819 | 23,819 | 2,620 | 23,819 | 23,819 |
| 2 | 1,640 | 14,908 | 38,726 | 1,640 | 14,908 | 38,726 |
| 3 | 1,409 | 12,812 | 51,538 | 1,409 | 12,812 | 51,538 |
| 4 | 1,102 | 10,016 | 61,555 | 1,102 | 10,016 | 61,555 |
| 5 | 1,007 | 9,150 | 70,705 | 1,007 | 9,150 | 70,705 |
| 6 | ,877 | 7,973 | 78,678 |  |  |  |
| 7 | ,669 | 6,078 | 84,756 |  |  |  |
| 8 | ,583 | 5,304 | 90,060 |  |  |  |
| 9 | ,466 | 4,240 | 94,300 |  |  |  |
| 10 | ,399 | 3,623 | 97,922 |  |  |  |
| 11 | ,229 | 2,078 | 100,000 |  |  |  |
| Extraction Method: Principal Component Analysis. | | | | | | |
| a. Only cases for which Lymphoma (0-1) = 1 are used in the analysis phase. | | | | | | |
